# Prediction models for neutralization activity against emerging SARS-CoV-2 variants: A cross-sectional study

**DOI:** 10.3389/fmicb.2023.1126527

**Published:** 2023-04-11

**Authors:** Atsushi Goto, Kei Miyakawa, Izumi Nakayama, Susumu Yagome, Juan Xu, Makoto Kaneko, Norihisa Ohtake, Hideaki Kato, Akihide Ryo

**Affiliations:** ^1^Department of Public Health, School of Medicine, Yokohama City University, Yokohama, Japan; ^2^Department of Health Data Science, Graduate School of Data Science, Yokohama City University, Yokohama, Japan; ^3^Department of Microbiology, Graduate School of Medicine, Yokohama City University, Yokohama, Japan; ^4^Center for Influenza and Respiratory Virus Research, National Institute of Infectious Diseases, Musashimurayama, Japan; ^5^Integrity Healthcare Co., Ltd., Tokyo, Japan; ^6^Department of Endocrinology and Metabolism, Graduate School of Medicine, Yokohama City University, Yokohama, Japan; ^7^Bioscience Division, Research and Development Department, Tosoh Corporation, Tokyo Research Center, Ayase, Japan; ^8^Infection Prevention and Control Department, Yokohama City University Hospital, Yokohama, Japan; ^9^Department of Virology III, National Institute of Infectious Diseases, Musashimurayama, Japan

**Keywords:** SARS-CoV-2, COVID-19, neutralizing antibody, Omicron sub-lineage, prediction model

## Abstract

**Objective:**

Despite extensive vaccination campaigns to combat the coronavirus disease (COVID-19) pandemic, variants of concern, particularly the Omicron variant (B.1.1.529 or BA.1), may escape the antibodies elicited by vaccination against SARS-CoV-2. Therefore, this study aimed to evaluate 50% neutralizing activity (NT_50_) against SARS-CoV-2 D614G, Delta, Omicron BA.1, and Omicron BA.2 and to develop prediction models to predict the risk of infection in a general population in Japan.

**Methods:**

We used a random 10% of samples from 1,277 participants in a population-based cross-sectional survey conducted in January and February 2022 in Yokohama City, the most populous municipality in Japan. We measured NT_50_ against D614G as a reference and three variants (Delta, Omicron BA.1, and BA.2) and immunoglobulin G against SARS-CoV-2 spike protein (SP-IgG).

**Results:**

Among 123 participants aged 20–74, 93% had received two doses of SARS-CoV-2 vaccine. The geometric means (95% confidence intervals) of NT_50_ were 65.5 (51.8–82.8) for D614G, 34.3 (27.1–43.4) for Delta, 14.9 (12.2–18.0) for Omicron BA.1, and 12.9 (11.3–14.7) for Omicron BA.2. The prediction model with SP-IgG titers for Omicron BA.1 performed better than the model for Omicron BA.2 (bias-corrected *R*^2^ with bootstrapping: 0.721 vs. 0.588). The models also performed better for BA.1 than for BA.2 (*R*^2^ = 0.850 vs. 0.150) in a validation study with 20 independent samples.

**Conclusion:**

In a general Japanese population with 93% of the population vaccinated with two doses of SARS-CoV-2 vaccine, neutralizing activity against Omicron BA.1 and BA.2 were substantially lower than those against D614G or the Delta variant. The prediction models for Omicron BA.1 and BA.2 showed moderate predictive ability and the model for BA.1 performed well in validation data.

## 1. Introduction

The coronavirus disease (COVID-19), first reported in Wuhan, Hubei Province, China, in December 2019, rapidly spread globally after March 2020, becoming a pandemic. Vaccination is the most effective strategy against COVID-19. Severe acute respiratory syndrome coronavirus-2 (SARS-CoV-2) vaccines, such as Pfizer-BioNTech BNT162b2, Moderna mRNA-1273, and AstraZeneca ChAdOx1-S, are currently being used worldwide (The Our World in Data, 2022a).

Almost all individuals in Japan who were interested in being vaccinated against COVID-19 received two doses of vaccine by the end of 2021 (Prime Minister’s Office of Japan, 2022). However, antibody titers decreased 6 months to 1 year after vaccination, resulting in individuals who had received two doses of vaccine being susceptible to infection (i.e., breakthrough infection) (Bergwerk et al., 2021; Yamamoto et al., 2022). Therefore, a third (booster) dose was administered in December 2021 6–8 months after administration of the second dose. In Japan, approximately 80% of the total population received at least two doses of SARS-CoV-2 vaccine, and approximately 66% received a third dose by October, 2022 (Prime Minister’s Office of Japan, 2022). This vaccination rate is similar to or higher than that in other high-income countries (The Our World in Data, 2022a). However, there is a concern that variants of concern (VOC), particularly the Omicron variant (B.1.1.529 or BA.1), could escape the antibodies elicited by vaccination against SARS-CoV-2 (Lu et al., 2022; Planas et al., 2022). Japan experienced the 6th and 7th waves of COVID-19 in 2022 (The Johns Hopkins Coronavirus Resource Center, 2022a) with Omicron variants becoming dominant (The Our World in Data, 2022b), despite most of the adult population receiving two doses of vaccines (The Johns Hopkins Coronavirus Resource Center, 2022b). Both Omicron BA.1 and BA.2 are known to evade immunity based on vaccination and/or prior infection (Planas et al., 2022; Yu et al., 2022b). Omicron variants carry a number of mutations in the spike protein of SARS-CoV-2, and BA.1 and BA.2 share many mutations in common (Viana et al., 2022). Previous studies with relatively small sample sizes have reported that neutralizing activity against Omicron BA.1 and BA.2 were suppressed when compared with those against Delta or D614G variant (Iketani et al., 2022; Uraki et al., 2022). Since August 2022, countries have started authorization of bivalent vaccines, also known as “updated boosters,” designed to protect against both the original strain of SARS-CoV-2 and lineages of the Omicron variant of SARS-CoV-2 (U.S. Food and Drug Administration, 2022). Since September 2022, bivalent vaccines designed to protect against both the original strain and the Omicron BA.1 subvariant have been administered in Japan (The Ministry of Health, Labour and Welfare, 2022).

As the “updated boosters” have become available (U.S. Food and Drug Administration, 2022), evaluation of neutralization activities against Omicron variants may help individuals make informed decisions regarding receiving the updated booster vaccine. However, quantitative measurements of neutralization against VOC are not publicly available and can only be performed in laboratories with advanced technology and experience in microbiology and immunology. Thus, prediction of neutralizing activity against VOC based on available clinical information including demographic factors, prior diagnosis of COVID-19, vaccination status, and antibody titers for immunoglobulin G against SARS-CoV-2 spike protein (SP-IgG) would be beneficial.

Therefore, in this population-based prevalence study in Yokohama City, the most populous municipality of Japan, we aimed to evaluate 50% neutralizing activity (NT_50_) against emerging immune escape variants such as Omicron BA.1 and BA.2. Furthermore, we aimed to develop and validate prediction models for NT_50_ against Omicron BA.1 and BA.2 by using clinical characteristics with and without IgG antibody titers for spike protein (SP-IgG) among randomly selected participants from a population-based study conducted in Yokohama, Japan.

## 2. Materials and methods

### 2.1. Study design and participants

In total, 6,000 residents were randomly selected from a Japanese population aged 20–74 years, from Yokohama City, Japan. With a population of approximately 3.7 million, Yokohama City is the most populated basic municipality in Japan and is located in Kanagawa Prefecture next to Tokyo. The study invitations were mailed to these residents in January 2022; 1,277 individuals (546 men and 731 women; response rate: 21.3%), with no confirmed diagnosis of COVID-19 within 2 weeks of the study entry, participated in this study from January 30 to February 28, 2022 (Supplementary Table 1 and Supplementary Figure 1). A random sample of 10% (*N* = 123) of the total study population was analyzed in this study. To validate the predictive performance, samples (*N* = 20) were randomly selected from the study population, without replacement (Supplementary Table 2). All participants provided written informed consent. The study was approved by the Institutional Review Board of the Yokohama City University.

### 2.2. Measurements

Each participant provided approximately 7 mL of blood and completed a questionnaire on prior COVID-19 diagnosis, vaccination against SARS-CoV-2, and lifestyle and social factor at study entry. Participants who provided incomplete answers, such as incorrect vaccination date, were supplemented with an additional questionnaire or underwent telephone interview. Therefore, there were no missing data in variables listed in Table 1.

**TABLE 1:** Baseline characteristics of participants (*n* = 123).

Characteristics	*N* = 123
Age (years)	54 (44, 65)
Male sex	54 (44%)
Prior diagnosis of COVID-19	6 (4.9%)
**Vaccination status**	
None	4 (3.3%)
Three	4 (3.3%)
Two	115 (93%)
Days since the last vaccination	180 (153, 202)
Unknown	4
**Coexisting conditions (number)**	
≥ 1	53 (43%)
None	70 (57%)
BMI	22.9 (21.0, 25.7)
**Smoking status**	
Non-smoker	93 (76%)
Occasional smoker	5 (4.1%)
Past smoker	15 (12%)
Regular smoker	10 (8.1%)
Passive smoking (≥once/week)	26 (21%)
**Alcohol drinking status**	
Non-drinker	56 (46%)
Occasional drinker	43 (35%)
Regular drinker	24 (20%)
**Sleeping time**	
<6 h	52 (42%)
≥6 h	71 (58%)
**Antibody measurements**	
SP-IgG (index)^1^	4.6 (3.8–5.7)
NP-total-Ig (index)^1^	0.1 (0.1–0.1)
NT_50_ for D614G^1^	65.5 (51.8–82.8)
NT_50_ for Delta^1^	34.3 (27.1–43.4)
NT_50_ for Omicron BA.1^1^	14.9 (12.2–18.0)
NT_50_ for Omicron BA.2^1^	12.9 (11.3–14.7)

Data are presented as median (interquartile range) or *n* (%), unless otherwise indicated.

NT_50_, neutralizing titers.

^1^Geometric mean (95 confidence interval).

Based on previous studies (Kubo et al., 2020; Goto et al., 2021; Miyakawa et al., 2021, 2022c; Kato et al., 2022a), levels of IgG antibodies against spike protein of the wild-type SARS-CoV-2 (SP-IgG) or total Ig antibodies against nucleocapsid protein of the wild-type SARS-CoV-2 (NP-total Ig) were measured in a laboratory at the Prime Health Partners Co., Ltd., (Yokohama, Japan) using a commercial chemiluminescent enzyme immunoassay (AIA-CL SARS-CoV-2 SP-IgG and NP-total Ig antibody detection reagents, Tosoh, Japan). An index ≥1.0 for these antibodies was considered positive according to the manufacturer’s instructions. The limit of detection (LOD) for SP-IgG and NP-total Ig was 0.1, and those with values below LOD were assigned a value of 0.05 for statistical analyses. For assessment of NT_50_ against emerging variants, a rapid quantitative neutralization test using HiBiT-tagged virus-like particle carrying the SARS-CoV-2 spike was performed at Yokohama City University as previously reported (Miyakawa et al., 2022b,c) using the spikes of D614G as a reference and three variants (Delta, Omicron BA.1, and BA.2). Briefly, VeroE6/TMPRSS2-LgBiT cells seeded in 96-well plates were inoculated with HiBiT-tagged virus-like particles containing diluted serum (1:20 to 1:43740 dilution). A total of 3 h after inoculation, the cells were washed and luciferase activity was measured using the GloMax Discover System (Promega). Serum was not removed during the incubation period. We have previously shown that the NT_50_ values from the rapid quantitative neutralization assay correlate with those from the assay using authentic viruses (Miyakawa et al., 2022a). The LOD for NT_50_ was 20, and those with values below LOD were assigned a value of 10 for statistical analyses.

### 2.3. Statistical analysis

Antibody titers were log (base 10)-transformed to improve normality assumptions in all analyses. To compare the distribution of NT_50_ between variants, geometric means (95% confidence intervals) were computed. Simple linear regression analyses were performed to examine associations of participants’ characteristics with log_10_(NT_50_) against each variant; multiple linear regression analyses were conducted to build prediction models for each variant. The group least absolute shrinkage and selection operator (LASSO) with standardization of all predictor variables was used to prevent overfitting and to consider grouped variables. The group LASSO estimates a set of regression coefficients that provide the lowest mean squared error in a five-fold cross-validation procedure The LASSO is conventionally used to perform variable selection and regularization (Tibshirani, 1996). When there are grouped variables, the group LASSO has been demonstrated to show better model performance than LASSO (Yuan and Lin, 2006; Sunandi et al., 2021). The oem package (version 2.0.10) in R (Xiong et al., 2016; Huling and Chien, 2022) was used to perform the group LASSO. Based on previous reports, the potential predictors analyzed included age (years), sex, prior diagnosis of COVID-19, vaccination status (none, two doses, or three doses; there was no participant with a single dose), coexisting conditions (≥1 or none), body mass index (BMI), smoking status (non-smoker, past smoker, occasional smoker, or regular smoker), passive smoking (≥once/week), alcohol drinking status (non-drinker, occasional drinker, or regular drinker), and sleep duration (<6 h or≥6 h) (Kato et al., 2022b; Lechmere et al., 2022; Lee et al., 2022). To improve model fit, continuous variables were transformed using a restricted cubic spline with three knots at the 10th, 50th, and 90th percentiles, if the non-linear relation between a continuous variable and the log_10_(NT_50_) was indicated by the model χ^2^ (Steyerberg, 2019). Standard regression diagnostics were performed while avoiding violation of regression assumptions (Fox and Weisberg, 2019). To evaluate the internal validity of the prediction model, we estimated Akaike’s Information Criterion (AIC) (Akaike, 1973) and performed 1,000 resampling procedures with replacement to obtain the bootstrap bias-corrected explained variation (i.e., *R*^2^). To further evaluate predictive performance in a validation dataset of 20 samples, we computed the predicted NT_50_ for BA.1 and BA.2 using the final prediction models and compared their values against the measured NT_50_. In the validation, the predictive ability was evaluated using the coefficient of determination (*R*^2^). All statistical analyses were performed with R version 4.1.2 (The R Foundation for Statistical Computing, Vienna, Austria).

## 3. Results

Among 123 participants (54 men and 69 women) aged 20–74 years, 93% received two doses (but not the third dose) of vaccine. The interval between the administration of the last dose of vaccine and data collection ranged from 0 to 286 days (median: 180 days; interquartile range: 153–202 days) (Table 1). Geometric means (95% confidence intervals) of SP-IgG (index) and NP-total-Ig (index) were 4.6 (3.8–5.7) and 0.1 (0.1–0.1), respectively.

**FIGURE 1:**
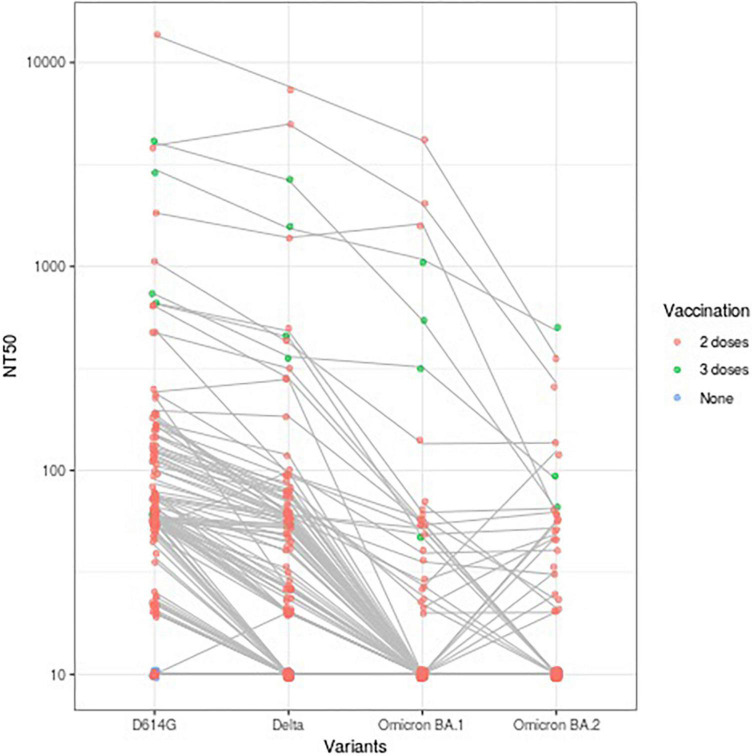
A total of 50% neutralizing activity (NT_50_) against variants of SARS-CoV-2 according to vaccination status (*n* = 123). NT_50_ against variants of SARS-CoV-2 of each participant are plotted and connected with lines. NT_50_: 50% neutralizing activity.

**FIGURE 2:**
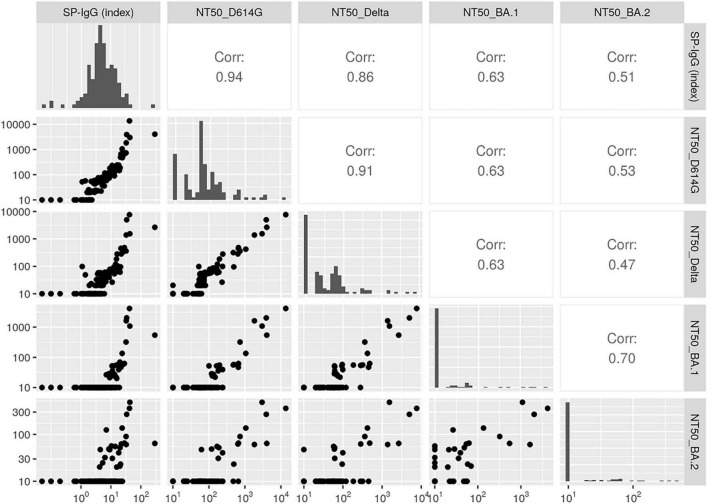
Correlations between immunoglobulin G against SARS-CoV-2 spike protein (SP-IgG) (index) and the 50% neutralizing activity (NT_50_) for variants (*n* = 123). Pairwise scatter plots **(lower left)**, Spearman’s correlations **(upper right)**, and histograms (diagonal elements) for antibody titers and NT_50_ are shown. In the scatter plots, the *X*-axis and *Y*-axis show values of antibody titers or NT_50_. In the histograms, the *X*-axis shows values of antibody titers or NT_50_ and the *Y*-axis shows frequency. Higher values represent a stronger correlation. NT_50_, 50% neutralizing activity; SP-IgG, immunoglobulin G against SARS-CoV-2 spike protein.

Neutralizing activity against Omicron BA.1 and BA.2 were more suppressed than those against D614G or Delta variant (geometric means [95% confidence intervals] of NT_50_: 65.5 [51.8–82.8] for D614G; 34.3 [27.1–43.4] for Delta; 14.9 [12.2–18.0] for Omicron BA.1; 12.9 [11.3–14.7] for Omicron BA.2) (Table 1 and Figure 1). Participants who had received three doses of vaccine (*n* = 4) showed similar patterns, but tended to have higher NT_50_ against all variants compared with participants who had received only two doses (*n* = 115). Moreover, the SP-IgG (index) strongly correlated with NT_50_ for D614G (*r* = 0.94) and Delta variant (*r* = 0.86) but modestly correlated with NT_50_ for Omicron BA.1 (*r* = 0.63) and BA.2 (*r* = 0.51) (Figure 2).

Table 2 shows associations of clinical characteristics and log-transformed SP-IgG (index) with log-transformed NT_50_ for Omicron BA.1. In univariate analyses, prior diagnosis of COVID-19, three doses of vaccines, and log-transformed SP-IgG were positively associated with NT_50_ for BA.1. In the model without SP-IgG, the final model with the group LASSO included age, prior diagnosis of COVID-19, and vaccination status. The model without SP-IgG had moderate predictive potential with adjusted *R*^2^ of 0.630 and bias-corrected *R*^2^ after bootstrapping of 0.559. The final model with SP-IgG included sex, prior diagnosis of COVID-19, vaccination status, and log-transformed SP-IgG had good predictive performance with adjusted *R*^2^ of 0.766 and bias-corrected *R*^2^ of 0.721.

**TABLE 2:** Associations of characteristics and antibody titers with NT_50_ for Omicron BA.1 (*n* = 123).

	Univariate		Model without SP-IgG		Model with SP-IgG	
**Characteristics**	**Beta**	**95% CI**	**Beta**	**95% CI**	**Beta**	**95% CI**
Age (per 10-year increment)	–0.03	–0.08, 0.03	–0.03	–0.06, 0.01		
Male sex	–0.11	–0.27, 0.06			0.04	–0.05, 0.13
Prior diagnosis of COVID-19	1.5	1.3, 1.8	1.6	1.3, 1.8	0.89	0.64, 1.1
**Vaccination status**						
None						
Three (vs. none)	1.1	0.43, 1.7	1.1	0.68, 1.5	0.87	0.37, 1.4
Two (vs. none)	0.15	–0.30, 0.59	0.11	–0.19, 0.40	0.39	–0.05, 0.83
**Coexisting conditions (number)**						
≥1						
None (vs. ≥1)	0.01	–0.16, 0.17				
BMI	–0.01	–0.03, 0.01				
**Smoking status**						
Non-smoker	..	..				
Occasional smoker (vs. non-smoker)	0.12	–0.31, 0.55				
Past smoker (vs. non-smoker)	–0.01	–0.27, 0.25				
Regular smoker (vs. non-smoker)	–0.12	–0.43, 0.19				
Passive smoking (≥once/week)	–0.13	–0.34, 0.07				
**Alcohol drinking status**						
Non-drinker						
Occasional drinker (vs. non-drinker)	0.01	–0.18, 0.19				
Regular drinker (vs. non-drinker)	0.10	–0.13, 0.32				
**Sleeping time**						
<6 h						
≥6 h	0.00	–0.17, 0.17				
**logSP-IgG**						
Restricted cubic spline: linear coefficient^1^	–0.26	–0.42,–0.09			–0.35	–0.61,–0.09
Restricted cubic spline: spline coefficient^1^	1.4	1.2, 1.7			1.1	0.77, 1.5
**Model performance**						
Adjusted *R*^2^			0.630		0.766	
Optimism-corrected *R*^2^			0.559		0.721	
AIC			49.4		–4.99	

NT_50_, neutralizing titers; CI, confidence interval; AIC, Akaike’s information criterion.

^1^A restricted cubic spline with three knots placed at 5th, 50th, and 95 percentiles was used. The first term corresponds to the linear coefficient and the second term corresponds to the cubic spline coefficient. In the model without SP-IgG, the predicted log_10_NT_50_ for BA.1 = 1.1113875 – 0.02912091 × age + 1.5696139 × prior COVID-19 + 1.0862965 × (vaccination = “Three doses”) + 0.10941138 × (vaccination = “Two doses”). In the model with SP-IgG, the predicted log_10_NT_50_ for BA.1 = 0.65209245 + 0.038310453 × gender + 0.89030215 × prior COVID-19 + 0.86544183 × (vaccination = “Three doses”) + 0.38826367 × (vaccination = “Two doses”) – 0.34790755 × logSP-IgG + 0.89243218 × (logSP-IgG– 0.15212068,0) _+_^3^– 1.6597735 × (logSP-IgG– 0.67209786) _+_^3^ + 0.7673413 × (logSP-IgG– 1.276841) _+_^3^. $x_{+}^{n}=\left\{ \begin{array}{c} x^{n}:x>0\\ 0:x\leq 0 \end{array}\right.$

Table 3 reports associations of clinical characteristics and log-transformed SP-IgG (index) with log-transformed NT_50_ for Omicron BA.2. In univariate analyses, prior diagnosis of COVID-19, three doses of vaccines, and log-transformed SP-IgG were positively associated with NT_50_ for BA.2. In the model without SP-IgG, the final model with the group LASSO included age, prior diagnosis of COVID-19, and vaccination status; the model had moderate predictive performance with adjusted *R*^2^ of 0.582 and bias-corrected *R*^2^ after bootstrapping of 0.497. The final model with SP-IgG included sex, prior diagnosis of COVID-19, vaccination status, and log-transformed SP-IgG had moderate predictive performance with adjusted *R*^2^ of 0.656 and bias-corrected *R*^2^ of 0.588.

**TABLE 3:** Associations of characteristics and antibody titers with NT_50_ for Omicron BA.2 (*n* = 123).

	Univariate		Model without SP-IgG		Model with SP-IgG	
**Characteristics**	**Beta**	**95% CI**	**Beta**	**Characteristics**	**Beta**	**95% CI**
Age (per 10-year increment)	–0.01	–0.05, 0.03	–0.01	–0.04, 0.01		
Male sex	–0.09	–0.20, 0.02			–0.01	–0.08, 0.07
Prior diagnosis of COVID-19	1.0	0.83, 1.2	1.0	0.87, 1.2	0.70	0.50, 0.90
**Vaccination status**						
None						
Three (vs. none)	0.66	0.23, 1.1	0.67	0.38, 1.0	0.59	0.18, 1.0
Two (vs. none)	0.09	–0.21, 0.40	0.06	–0.16, 0.27	0.22	–0.14, 0.59
**Coexisting conditions (number)**						
≥1						
None (vs. ≥1)	0.01	–0.11, 0.12				
BMI	0.00	–0.02, 0.01				
**Smoking status**						
Non-smoker						
Occasional smoker (vs. non-smoker)	0.08	–0.21, 0.37				
Past smoker (vs. non-smoker)	0.00	–0.18, 0.18				
Regular smoker (vs. non-smoker)	–0.06	–0.27, 0.15				
Passive smoking (≥once/week)	–0.08	–0.22, 0.06				
**Alcohol drinking status**						
Non-drinker	..					
Occasional drinker (vs. non-drinker)	–0.02	–0.15, 0.11				
Regular drinker (vs. non-drinker)	0.06	–0.09, 0.22				
**Sleeping time**						
<6 h	..					
≥6 h	–0.03	–0.14, 0.09				
**logSP-IgG**						
Restricted cubic spline: linear coefficient^1^	–0.15	–0.29,–0.02			–0.18	–0.40, 0.03
Restricted cubic spline: spline coefficient^1^	0.88	0.67, 1.1			0.57	0.28, 0.87
**Model performance**						
Adjusted *R*^2^			0.582		0.656	
Optimism-corrected *R*^2^			0.497		0.588	
AIC			–29.7		–51.9	

NT_50_, neutralizing titers; CI, confidence interval; AIC, Akaike’s information criterion.

^1^A restricted cubic spline with three knots placed at 5th, 50th, and 95 percentiles was used. The first term corresponds to the linear coefficient and the second term corresponds to the cubic spline coefficient. In the model without SP-IgG, the predicted log_10_NT_50_ for BA.2 = 1.04732 – 0.012371236 × age + 1.0477396 × prior COVID-19 + 0.67405646 × (vaccination = “Three doses”) + 0.058003678 × (vaccination = “Two doses”). In the model with SP-IgG, the predicted log_10_NT_50_ for BA.2 = 0.81802988 – 0.0061103021 × gender + 0.7000916 × prior COVID-19 + 0.59005683 × (vaccination = “Three doses”) + 0.22412643 × (vaccination = “Two doses”) – 0.18197012 × logSP-IgG + 0.45275045 × (logSP-IgG– 0.15212068,0) _+_^3^– 0.84203956 × (logSP-IgG– 0.67209786,0) _+_^3^ + 0.3892891 × (logSP-IgG– 1.276841,0) _+_^3^. $x_{+}^{n}=\left\{ \begin{array}{c} x^{n}:x>0\\ 0:x\leq 0 \end{array}\right.$

**FIGURE 3:**
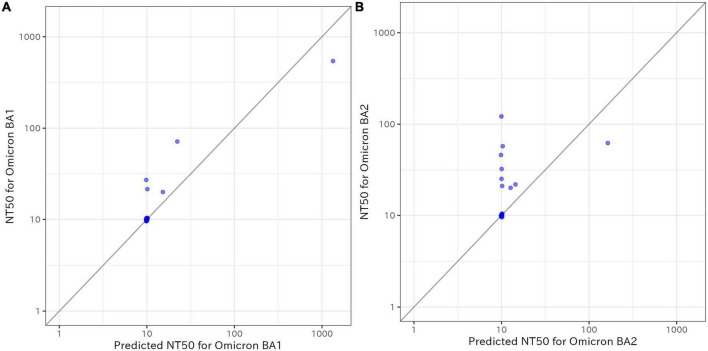
Measured 50% neutralizing activity (NT_50_) for Omicron BA.1 and BA.2 compared with the predicted values from the final models using immunoglobulin G against SARS-CoV-2 spike protein SP-IgG in the validation study (*n* = 20). **(A)** Results for BA.1, **(B)** results for BA.2. The *X*-axis shows the predicted values and the *Y*-axis shows the values of NT_50_. The coefficient of determination (*R*^2^) was 0.850 for BA.1 and 0.150 for BA.2. NT_50_: 50% neutralizing activity; SP-IgG: immunoglobulin G against SARS-CoV-2 spike protein.

Figure 3 shows the measured NT_50_ for Omicron BA.1 and BA.2 compared with the predicted values from the final models using SP-IgG in the validation study. BA.1 had an acceptable *R*^2^ and the predicted values for BA.1 slightly underestimated the NT_50_; however, the model for BA.2 severely underestimated the NT_50_ (*R*^2^ = 0.850 for BA.1 vs. 0.150 for BA.2).

## 4. Discussion

In this population-based cross-sectional study in Yokohama City, Japan, we evaluated NT_50_ against D614G, Delta, Omicron BA.1, and Omicron BA.2 variants of SARS-CoV-2 and developed prediction models for them for a general population in Japan. We found that NT50 against Omicron BA.1 and Omicron BA.2 were more substantially suppressed than that against D614G or Delta variant, highlighting the population-level insufficient humoral immunity against Omicron variants. Moreover, SP-IgG (index) more strongly correlated with NT_50_ against Omicron BA.1 than against Omicron BA.2. The prediction model with SP-IgG (index) had satisfactory performance against NT_50_ for Omicron BA.1 and performed well in the validation sample. The prediction model for Omicron BA.2 showed moderate predictive performance against NT_50_, but its predictive ability was poor in the validation sample. To the best of our knowledge, this is the first study to develop prediction models of NT_50_ against Omicron BA.1 and BA.2. The prediction models developed may be helpful in knowing the humoral immunity against Omicron variants.

Our findings demonstrating suppressed neutralizing activity against both the Omicron BA.1 and BA.2 variants suggest that the second dose of vaccine is not sufficient in most individuals to neutralize these variants. The similar neutralizing activity against BA.1 and BA.2 variants observed in our study are in accordance with previous reports (Yu et al., 2022a).

Our prediction models showed moderate predictive ability for NT_50_ against Omicron BA.1 and BA.2, with better performance for BA.1 than for BA.2. Despite expectations at the beginning of the COVID-19 pandemic, antibody measurements against SARS-CoV-2 have not been widely used in real-world settings. Because the “updated boosters” against the original strain and Omicron variants of SARS-CoV-2 have become available (The Ministry of Health, Labour and Welfare, 2022; U.S. Food and Drug Administration, 2022), utilizing these prediction models would help people predict their humoral immunity against Omicron variants, possibly facilitating decision-making about receiving the “updated boosters.” Our prediction models could be used to predict the risk of viral infection, although prospective cohort studies are required to confirm our findings.

For Omicron BA.1, the prediction model with SP-IgG (index) performed well; however, for Omicron BA.2, the prediction model did not perform well. This discrepancy may be partially explained by the deviation in the correlation coefficients with NT_50_ against Omicron variants (Omicron BA.1: *r* = 0.63; BA.2: *r* = 0.51). Further, two groups of people with high SP-IgG titers could be characterized: those with a minimal NT_50_ and those with relatively high NT_50_ against Omicron variants (Figure 2). However, we were unable to find factors that could explain these differences (Tables 2, 3). In order to build more accurate prediction models, it may be necessary to develop SP-IgG titers against the variant spikes, rather than the spike of the wild-type SARS-CoV-2.

The strengths of this study include its population-based design with detailed assessments of demographic data, vaccination status, and previous diagnosis of COVID-19. In addition, our developed prediction models were validated in an independent sample. However, the present study has some limitations. First, the cross-sectional nature of the study limits the ability to investigate causality between measured variables. However, based on the growing evidence of the effectiveness of vaccination on immunity against COVID-19, our findings provide an accurate description of the humoral immunity against variants, including assessments of the emerging variants such as Omicron BA.1 and BA.2. Second, the response rate of our study was approximately 21%, which is not sufficiently high. Last, our study provides evidence regarding a certain period in Yokohama City, Japan, and generalizability may be limited. Importantly, none of the participants in our study population received a bivalent vaccine, and the majority of participants had received only two doses of vaccine. Because of this, the timeline of our study limits the applicability of our prediction models.

To summarize, in this population-based cross-sectional study conducted in Japan, neutralizing activity against Omicron BA.1 and BA.2 was substantially reduced compared with that of the D614G variant, and newly developed prediction models that used clinical information and SP-IgG had moderate ability to predict the NT_50_ for Omicron BA1 and BA.2.

## Data availability statement

The datasets presented in this article are not readily available because the information could compromise the privacy of participants. Requests to access the datasets should be directed to AG, agoto@yokohama-cu.ac.jp.

## Ethics statement

The studies involving human participants were reviewed and approved by the Yokohama City University. The patients/participants provided their written informed consent to participate in this study.

## Author contributions

AG contributed to the study design, data collection, statistical analysis and interpretation of data, and the drafting and editing of the manuscript. KM, NO, and HK contributed to the study design, laboratory testing, and interpretation of data. IN, SY, JX, and MK contributed to the study design, data collection, and interpretation of data. AR contributed to the study design, data collection, and supervised the analysis and preparation of the manuscript. All the authors made critical revisions to the manuscript for important intellectual content and approved the final manuscript.

## Supplementary material

Supplementary Figure 1 The SP-IgG index in the source population according to vaccination status (*N* = 1,277). The geometric mean titer of the SP-IgG index (95% confidence interval) is shown.
