# Supplementary material for: Prediction models for neutralization activity against emerging SARS-CoV-2 variants: A cross-sectional study
Source: Front Microbiol. 2023 Apr 11;14:1126527. doi: 10.3389/fmicb.2023.1126527 (PMC10126441; doi:10.3389/fmicb.2023.1126527)
Supplement: Supplementary file 1 [file Table_1.DOCX]

**Supplementary Table 1. Baseline characteristics of a source population (N = 1,277).**

| Characteristic | N = 1,277^1^ |
| --- | --- |
| Age (years) | 51 (40, 60) |
| Male gender | 546 (43%) |
| Prior diagnosis of COVID-19 | 36 (2.8%) |
| Vaccination status |  |
| None | 43 (3.4%) |
| Single | 3 (0.2%) |
| Two | 1,152 (90%) |
| Three | 79 (6.2%) |
| Days since the last vaccination | 167 (136, 195) |
| Not applicable (unvaccinated) | 43 |
| Coexisting conditions (number) |  |
| ≥1 | 496 (39%) |
| None | 781 (61%) |
| BMI | 22.3 (20.4, 24.8) |
| Unknown | 2 |
| Smoking status |  |
| Non-smoker | 952 (75%) |
| Past smoker | 161 (13%) |
| Occasional smoker | 18 (1.4%) |
| Regular smoker | 146 (11%) |
| Alcohol drinking status |  |
| Non-drinker | 535 (42%) |
| Occasional drinker | 447 (35%) |
| Regular drinker | 295 (23%) |

Data are presented as median (interquartile range) or n (%), unless otherwise indicated.

**Supplementary Table 2. Baseline characteristics of samples in a validation study (N = 20).**

| Characteristic | N = 20^1^ |
| --- | --- |
| Age (years) | 46 (38, 59) |
| Male gender | 9 (45%) |
| Prior diagnosis of COVID-19 | 0 (0%) |
| Vaccination status |  |
| Two | 19 (95%) |
| Three | 1 (5.0%) |
| Days since the last vaccination | 160 (136, 194) |
| Coexisting conditions (number) |  |
| ≥1 | 3 (15%) |
| None | 17 (85%) |
| BMI | 22.46 (20.60, 25.88) |
| Smoking status |  |
| Non-smoker | 17 (85%) |
| Past smoker | 2 (10%) |
| Regular smoker | 1 (5.0%) |
| Passive smoking (≥ once/week) | 3 (15%) |
| Alcohol drinking status |  |
| Non-drinker | 12 (60%) |
| Occasional drinker | 7 (35%) |
| Regular drinker | 1 (5.0%) |
|  | |

Data are presented as median (interquartile range) or n (%), unless otherwise indicated.
