# Supplementary figures and images for: Prediction models for neutralization activity against emerging SARS-CoV-2 variants: A cross-sectional study
Source: Front Microbiol. 2023 Apr 11;14:1126527. doi: 10.3389/fmicb.2023.1126527 (PMC10126441; doi:10.3389/fmicb.2023.1126527)

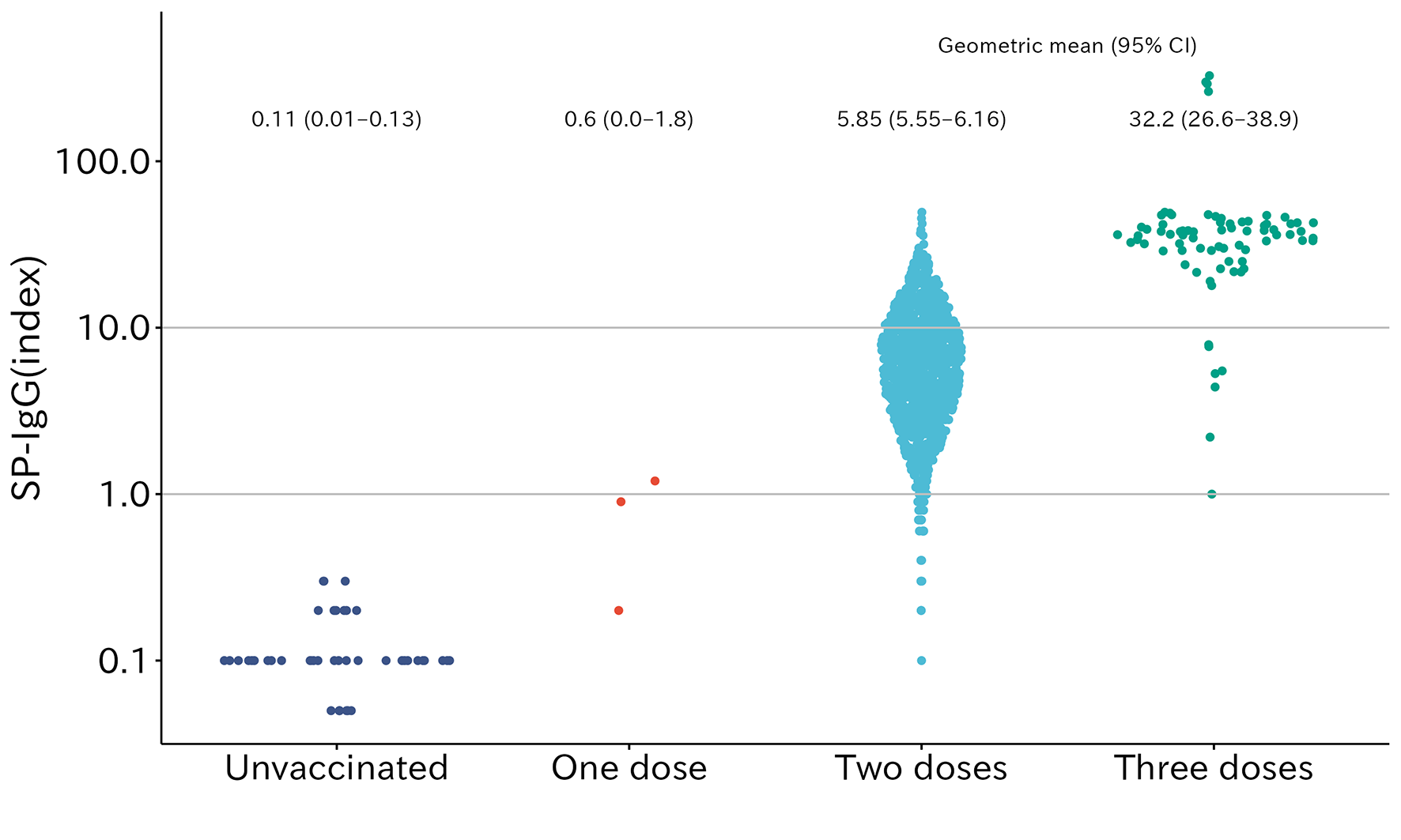

Supplement: Supplementary Figure 1 — The SP-IgG index in the source population according to vaccination status (N = 1,277). The geometric mean titer of the SP-IgG index (95% confidence interval) is shown. [file Image_1.TIFF]
